# Preparation of Micro/Nano-Structure Copper-Substituted Hydroxyapatite Scaffolds with Improved Angiogenesis Capacity for Bone Regeneration

**DOI:** 10.3390/ma11091516

**Published:** 2018-08-23

**Authors:** Adil Elrayah, Wei Zhi, Shi Feng, Salih Al-Ezzi, He Lei, Jie Weng

**Affiliations:** 1 Key Laboratory of Advanced Technologies of Materials (MOE), School of Materials Science and Engineering, Southwest Jiaotong University, Chengdu 610031, China; adil.karary@yahoo.com (A.E.); Zhiwei@home.swjtu.edu.cn (W.Z.); salihlink@yahoo.com (S.A.-E.); scuhelei@outlook.com (H.L.); 2 General Science Directorate, Karary University, Omdurman 12304, Sudan; 3 Collaboration Innovation Center for Tissue Repair Material Engineering Technology, China West Normal University, Nanchong 637002, China; shifeng@cwnu.edu.cn

**Keywords:** hydroxyapatite, hydrothermal, copper ions, micro/nano-structure, angiogenesis

## Abstract

The surface microstructures of calcium phosphate ceramics play an essential role in determining bone regeneration. However, it is difficult to produce micro/nano-structures on the surface of the porous hydroxyapatite (HA) scaffolds. In this study, we successfully developed and fabricated various micro/nano-structured surfaces on the HA scaffolds in copper ion (Cu^2+^)-containing solutions under hydrothermal conditions. The micro/nano-structures on the surface of the HA scaffolds were controlled by modulating the Cu^2+^ concentrations during the hydrothermal process. With an increase in the Cu^2+^ concentration, the surface morphology of the HA scaffolds changed significantly from sphere-like to flower-like, before becoming nano-structures. These findings indicated that the Cu^2+^ concentration affects the morphologies of calcium phosphate coatings that grow on the HA scaffolds. In vitro endothelial cell (EC) cultures showed that the cell proliferation was significantly enhanced when cultured on the flower-like morphology compared with other morphologies. Furthermore, an in vivo test in New Zealand rabbits demonstrated that the HA scaffold with the flower-like surface resulted in more angiogenesis compared with the control scaffold. This copper-assisted hydrothermal deposition process provides a simple and controllable route for engineering a micro/nano-structured surface on the HA scaffolds, which has benefits in terms of angiogenesis and bone regeneration.

## 1. Introduction:

As the common mineral component of human bones, hydroxyapatite (HA, Ca_10_(PO_4_)_6_(OH)_2_) has been widely used in bone repair due to its excellent biocompatibility and bioactivity [1]. Many studies have shown that the surface properties of bone scaffolds are important for cell response and tissue formation [2]. The surface microstructure of calcium phosphate has been proven to affect cell attachment, proliferation and differentiation [3]. In addition to the hierarchical micro/nano-structure of bone, the mineral in human bone is not pure stoichiometric HA as it is partially substituted by elements, such as Na, K, Sr, Cu and F [4]. The substituted elements not only affect the physical structure of apatite, but also strengthen its biological function [5]. Therefore, mimicking bone structures is a good choice for scaffold design.

Bone healing involves a series of biological events, such as inflammatory reactions, vascularization and osteogenesis. Chim et al. stated that angiogenesis is a key process underlying the repair of bone defects [6]. Failures in the restoration of vasculature or the lack of angiogenesis usually leads to delayed healing or ongoing restorative failure [7]. Particularly, the in vivo implantation failure is largely due to a lack of angiogenesis in the scaffold [8]. Traditionally, the synthesized HA biomaterials are often used for the replacement and regeneration of hard tissues, such as bones and teeth [9], without consideration of angiogenesis. The lack of angiogenesis delays osteoid deposition and matrix development [7], while it also decreases the bone healing rate [10]. To address these challenges, the HA scaffolds with desirable micro/nano-structures need to be fabricated for regulating cell behavior. Thus, various techniques have been reported for the creation of surface coatings or modification of calcium phosphate scaffolds, such as precipitation, sol-gel, solid-state, hydrothermal and biomimetic methods [9,11,12]. Of these, the hydrothermal process provides quick production with relative technical simplicity and high product crystallinity [9]. In the hydrothermal method, the pH and ion concentrations (e.g., Ca^2+^, PO_4_^3−^) are the key parameters affecting the morphology and the crystal size of formed coatings [13]. Thus, this method exhibits promising advantages in the preparation of micro/nano-structured HA scaffolds as it can control surface morphology regardless of the scaffold shape [14].

However, potential strategies should be explored to enhance the angiogenesis capacity of the HA scaffolds. Copper acting as a trace element in the human body plays a vital role in the angiogenic process and promotes endothelial cell migration [15]. Therefore, Cu^2+^ was doped into HA powders or adsorbed into the calcium phosphate scaffold for increasing the angiogenesis capacity. Barralet et al. [16] found that low doses of Cu^2+^ adsorbed into the calcium phosphate scaffold led to the formation of micro vessels along the macro pore axis, whereas Xiao et al. [11] proposed that Cu^2+^ could affect HA morphogenesis under hydrothermal conditions. Therefore, the addition of Cu^2+^ may not only influence the physical and chemical properties of HA, but also strengthen its bioactivity [17]. However, these works did not investigate if Cu^2+^ could be applied to regulate the surface morphology of the HA scaffolds under hydrothermal conditions. Among the available literature about the analysis of HA coatings in hydrothermal processes, there is still a lack of understanding regarding the effect of Cu^2+^.

Our previous study controlled the nanostructures of the calcium phosphate coatings that were hydrothermally deposited on the HA scaffolds by introducing organic crystal growth modifiers, such as inositol hexacarboxylic acid (H_6_L) [11]. As many organic modifiers (e.g., H_6_L) are not present in the human body, their use might raise concerns about safety and clinical effects. To eliminate these drawbacks, inorganic Cu^2+^ might be substituted hydrothermally in HA to produce the desired micro/nano-structure and stimulate angiogenesis. However, no study has investigated the impact of Cu^2+^ on the HA fiber scaffold morphologies for enhancing the angiogenesis response.

The objective of this research was to develop micro/nano-structured HA scaffolds with improved angiogenesis capacity for enhancing bone regeneration. As mentioned before, we hypothesize that the addition of Cu^2+^ would affect the surface nano-structures of the HA scaffold under hydrothermal conditions and enhance angiogenesis. To provide empirical evidence for this hypothesis, a copper ion-containing aqueous solution was used to regulate the surface properties of HA scaffolds. Additionally, the angiogenesis capacity of the copper-substituted HA scaffolds was investigated both in vitro and in vivo.

## 2. Material and Methods

### 2.1. Preparation of HA Scaffolds

The HA slurry was prepared by mixing 20 g of HA powder (Kelong, Chemical, Chengdu, China) with 100 mL of sodium alginate solution (3 wt %) [18]. After this, the slurry was transferred into a syringe and injected into a CaCl_2_ solution (0.2 mol/L) to form cross-linked fibers. The fibers were immediately collected and packed into molds to create cylindrical scaffolds. The cylindrical scaffolds were dried at 70 °C for 10 h and sintered at 1200 °C for 44 h [18]. The fabricated scaffolds have dimensions of 5 × 10 mm.

### 2.2. Hydrothermal Construction of Micro-Nano-Structured Surface on HA Scaffolds

**Figure 1:**
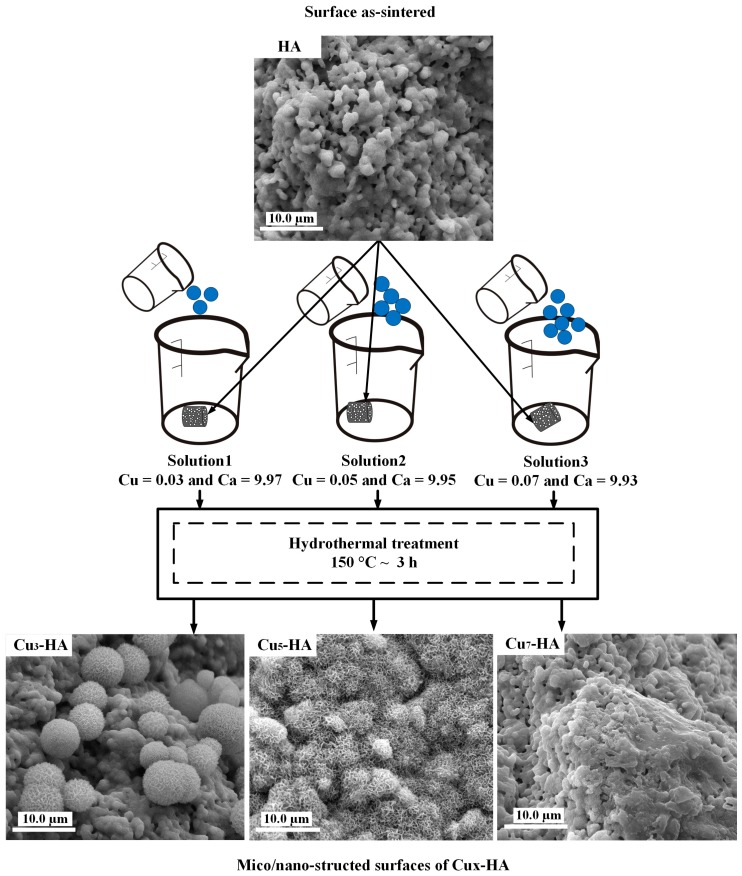
The experimental process used to hydrothermally construct the micro-nano-structured surface on hydroxyapatite (HA) fiber scaffolds.

The copper-substituted hydroxyapatites are represented as Cu_*x*−_HA, where *x* is the percentage of Cu/(Ca + Cu) = *x* (%) with a general formula of Ca_(10−*x*)_Cu_*x*_(PO_4_)_6_(OH)_2_ as shown in Equation (1). Hydrothermal conditions (150 °C; ~3 h) were used to fabricate three series groups of HA scaffolds, which were named Cu_3_-HA, Cu_5_-HA and Cu_7_-HA. Three types of initial solutions with a molar ratio of (Ca + Cu)/P of 1.67 were applied. Solution (1) included 0.097 mol/L Ca(NO_3_)_2_, 0.003 mol/L Cu(NO_3_)_2_ and 0.06 mol/L Na_2_HPO_4_. The molar ratio of Cu/(Ca + Cu) was 3% and Ca/P was 1.62. Solution (2) contained 0.095 mol/L Ca(NO_3_)_2_, 0.005 mol/L Cu(NO_3_)_2_ and 0.06 mol/L Na_2_HPO_4_. For this solution, the molar ratio of Cu/(Ca + Cu) was 5% and Ca/P was 1.58. Solution (3) included 0.093 mol/L Ca(NO_3_)_2_, 0.007 mol/L Cu(NO_3_)_2_ and 0.06 mol/L Na_2_HPO_4_. In this solution, the molar ratio of Cu/(Ca + Cu) was 7% and Ca/P was 1.55, which is shown in Table 1. Each solution was adjusted to have a pH of 2.50 ± 0.05 by dropwise addition of HNO_3_. Additionally, 1 g of urea (CH_4_N_2_O) was added to increase the super saturation and support the elongation of HA crystals [19]. The as-sintered HA scaffolds, i.e., Solutions (1), (2) and (3), were immersed in three containers. After this, the scaffolds were heated in an autoclave to 150 °C for 3 h. Later, they were harvested and rinsed with deionized water, before finally being dried in an autoclave at 80 °C for 3 h [11]. Figure 1 illustrates the hydrothermal experimental process applied to construct the micro-nano-surface on the HA fiber scaffolds with 3 solutions.

(1) $$\left(10-x\right){\mathrm{Ca}}^{2+}+x{\mathrm{Cu}}^{2+}+6{{\mathrm{PO}}_{4}}^{3-}+2H_{2}O\to {{\mathrm{Ca}}^{2+}}_{\left(10-x\right)}{{\mathrm{Cu}}^{2+}}_{x}{\left({\mathrm{PO}}_{4}\right)}_{6}{\left(\mathrm{OH}\right)}_{2}+2H^{+}$$

### 2.3. In Vitro Cell Culture on HA and Cu_x_-HA Scaffolds

Human endothelial cells (ECs) were obtained from Sichuan University and cultured in α-minimum essential medium (MEM), which was supplemented with 10% fetal bovine serum (FBS) and 1% penicillin/streptomycin (37 °C, 5% CO_2_ and 95% humidified air). The scaffolds were sterilized at 121 °C for 30 min, before being placed into 24-well culture plates. The ECs (passage 4) were seeded onto the series groups of scaffolds at a density of 1 × 10^5^ cells/scaffold, before being cultured in a media that was supplemented with 50 nmol/L L-ascorbic acid, 10 mmol/L β-glycerophosphate and 0.1 µmol/L dexamethasone. The cultured media were refreshed every 2 days. The adhesion and spreading of the EC on the surface of scaffolds were observed by scanning electronic microscopy (SEM). The cells were fixed with incubation in PBS for 5 min, before being rinsed with PBS to remove the excess dyed cells. The fixed cells were cleaned with the following sequence of alcohol solutions: 70%, 80%, 90% and finally 100%.

The viability of EC was determined by Alamar Blue (AB) assays. At 1, 3 and 5 days, the medium was removed and 300 µL of AB reagent (10% Alamar blue, 80% media and 10% FBS; *v*/*v*) was added to each well and incubated at 37 °C for 3 h. The wells without the cells were used as the blank controls. After this, 200 µL of the supernatant fluid was pipetted into a 24-well plate at a wavelength of 570 nm (excitation) or 600 nm (emission) with an ELISA micro plate reader (Molecular Devices, Sunnyvale, CA, USA). Statistical analysis was performed using the t-test method, while comparison tests were used to evaluate the different groups of scaffolds. The level of significance was set at *p* < 0.05.

### 2.4. Rabbit Model and Surgical Procedures

**Figure 2:**
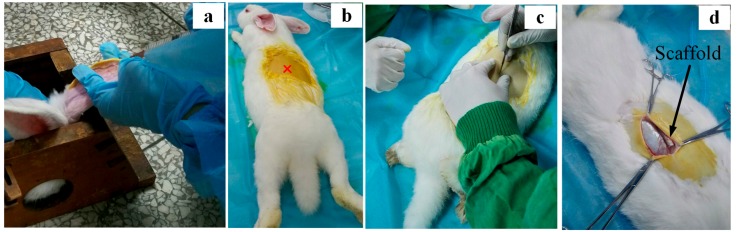
Pre-surgical operations of New Zealand rabbits. (**a**) Surgical operation under general anesthesia by a lateral ear vein injection; (**b**) implantation location; (**c**) start of surgery operation to open skin; and (**d**) subcutaneous socket with implantation of scaffolds.

Six adult New Zealand white rabbits (female, 80 days old and weight of 2.5 ± 0.6 kg) were used in this test. After obtaining the permission of the local animal care committee (Animal Center, Sichuan University), HA and Cu_5_-HA fiber scaffolds (9 scaffolds of each type) were implanted subcutaneously for 1, 4 and 8 weeks. Figure 2b illustrates the poor vascular implantation location inside the skeleton skin that is close to spine tissues. The surgical operation was performed under general anesthesia by a lateral ear vein injection of sodium pentobarbital (40 mg/kg body weight) and sterile conditions, which is shown in Figure 2. Before the surgical operation started, all tools and materials were sterilized. After this, pentobarbital was prepared using 0.6 g of pentobarbital drug with 15 mL of 3% saline solution (NaCl). The operation room was sterilized by ultraviolet (UV) light for 30 min. After the surgeries, 0.20 g/kg penicillin was intramuscularly injected for five consecutive days to prevent infection. After the operation, the animals were fully weight bearing and received a normal diet.

### 2.5. Harvesting of the Implanted Scaffolds

The animals were sacrificed with a celiac injection of an excessive amount of pentobarbital sodium after 1, 4 and 8 weeks. After this, the scaffolds were harvested along with the surrounding tissues and fixed in 500 mL of 10% buffered formaldehyde. The formalin was changed every 3 days at room temperature for 1 week. After this, the scaffolds were washed with dynamic flowing water for 24 h. Afterwards, the scaffolds were further cleaned with different concentrations (i.e., 70%, 80%, 90%, 95% and 100%) of alcohol. Finally, the scaffolds were embedded with the powder metallurgical metallographic rubber curing agents (PMMA), which were obtained from Brand SPK (Chengdu, China). The embedded scaffolds were sectioned with an average thickness of 300 µm and embedded using a microtome (Sp-1600’, Leica, Germany), which was equipped with a diamond saw blade attached to a knurled screw to control the thickness of the section.

## 3. Results and Discussion

### 3.1. Micro/Nano-Structured Surfaces of HA and Cu_x_-HA Scaffolds

**Figure 3:**
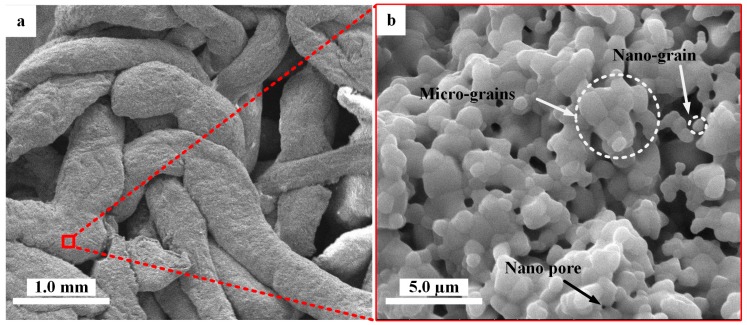
Scanning electron microscopy (SEM) photographs of the typical morphology of as-sintered HA scaffolds: (**a**) typical morphology of HA fiber scaffold and (**b**) the surface of HA scaffold fiber with micropores.

We investigated the formation of micro/nano-structures on the HA scaffolds under hydrothermal conditions. First, the hydrothermal aqueous solutions with different Cu concentrations were prepared as shown in Table 1. After this, the as-sintered HA scaffolds with smooth crystalline grains and micro/nano-pores were soaked in different aqueous solutions, before being placed into the autoclaves. The scaffolds were treated under the following initial conditions: three hours, pH of 2.3 and 150 °C. After the reaction continued for three hours, many new micro/nano-crystals formed on the surfaces of the scaffolds. The micro/nano-structure surfaces of the HA scaffolds were subsequently characterized (Figure 3). The surface of the as-sintered HA scaffold (Figure 3a) was composed of smooth crystalline grains, which showed micro/nano-pores as indicated with white arrows and circles in Figure 3b.

**Figure 4:**
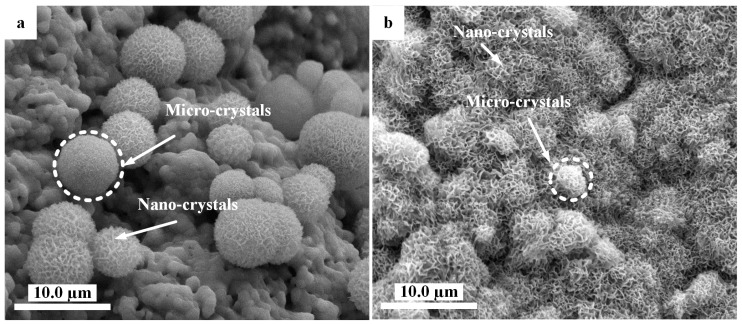

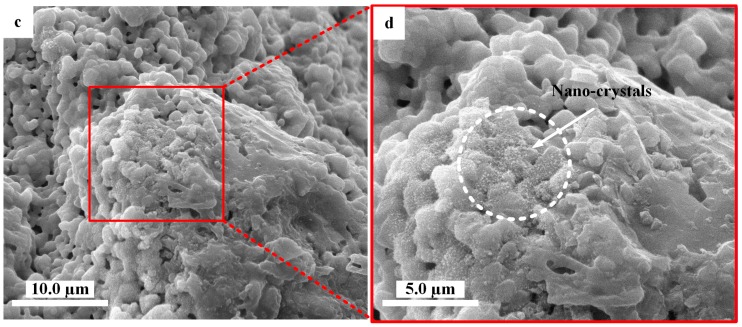
SEM photographs showing morphologies on HA scaffolds with the assistance of hydrothermal conditions. (**a**) Microspheres grown on a Cu_3_-HA scaffold; (**b**) micro/nano-structured surface with flower-like crystals on Cu_5_-HA scaffolds; and (**c**,**d**) nano-structured surface on Cu_7_-HA scaffolds.

When the HA scaffolds were immersed in solutions under hydrothermal conditions, the surface of the HA scaffolds changed remarkably to have different morphologies. Figure 4 illustrated the microspheres containing nano-sheet crystals with an average diameter of 6.7 ± 0.8 µm, which were obtained on the Cu_3_-HA scaffold in Solution (1) (Figure 4a). Flower-like micro/nano-crystals with an average diameter of 3.2 ± 0.5 µm formed the full coating on the surface of the Cu_5_-HA scaffolds with further increases in copper in Solution (2), which is shown in Figure 4b.

Nano-crystals formed on the surface of the Cu_7_-HA scaffolds in Solution (3) when adding the maximum amount of copper ions (Figure 4c,d). From the above results, the addition of copper could regulate the micro/nano-structures of the HA fiber scaffolds surfaces during hydrothermal depositions. With increasing amounts of copper ions in the solutions, the surface morphologies of the scaffolds tended to change from sphere-like to flower-like, before becoming nano-structured.

**Figure 5:**
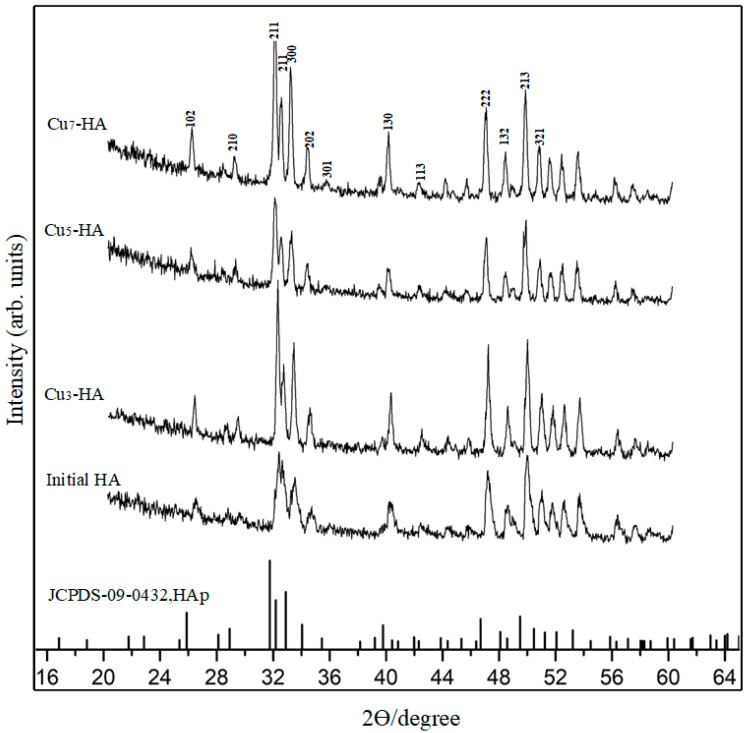
X-ray diffraction (XRD) spectra of HA, Cu_3_-HA, Cu_5_-HA, Cu_7_-HA scaffolds and standard spectra of HAp.

Furthermore, the scaffolds were analyzed using energy dispersive X-ray (EDX, PANalytical B.V., Almelo, Holland) spectroscopy and X-ray diffraction (XRD). EDX analyses of the Cu_5_-HA and Cu_7_-HA scaffold surfaces showed peaks for Ca, Cu, P and O, with a Ca/P ratio of 1.63 and 1.58, respectively. This suggests a Ca-deficient apatite phase. EDX analyses of the Cu_3_-HA and HA scaffold surfaces showed Ca/P ratios of 1.61 and 1.68, respectively. However, no copper element was observed in the Cu_3_-HA scaffolds, which may be due to the lower amount of copper in the initial solutions. To further characterize the surface composition of these scaffolds, we used X-ray diffraction (XRD, PANalytical X’Pert PRO, Philips, Netherlands, CuKα, 35 mA, 45 kV). The XRD patterns (Figure 5) demonstrated that all samples were composed of a HA phase that is similar to the phase constitution of an untreated initial HA scaffold after 3 h of hydrothermal treatment. Furthermore, no significant difference was observed for all Cu_x_-HA samples with respect to the concentration of copper ions, which may be attributed to the strong diffraction peak of the substrate.

**Figure 6:**
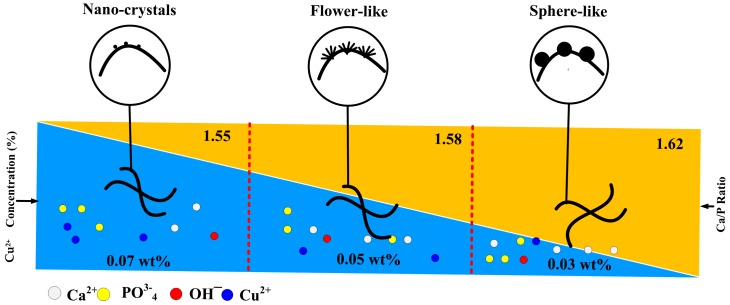
The mechanism and correlations controlling the micro/nano-structure of Cu_x_-HA scaffolds by hydrothermal reaction.

In general, the growth of calcium phosphate depended on the solution pH, the temperature, the Ca/P ratio and the presence of ions or biomolecular [20]. In this study, calcium phosphate crystals grew on the surface of the HA scaffolds when adding copper ions to the hydrothermal solution, which suggested that copper ions played a key role in the process of calcium phosphate deposition under hydrothermal conditions. Based on the above results, we predicted the mechanism behind calcium phosphate growth that occurs with the assistance of copper ions, which is shown in Figure 6. On one hand, when the scaffolds were immersed into solutions, under the initial acidic conditions (pH = 2.5), Ca^2+^ and PO_4_^3−^ probably dissolved into the surrounding solution from the scaffolds. With an increase in the reaction temperature, the solution became increasingly alkaline because of urea decomposition. The dissolved ions may accelerate crystal nucleation around the surface of the scaffolds. On the other hand, the addition of copper ions resulted in the formation of calcium phosphate crystals on the HA scaffold. When the added amount of copper ions was 0.03 wt %, the sphere-like calcium phosphate crystals formed on the scaffold surface, while the flower-like calcium phosphate coatings formed when the added amount of copper ions was 0.05 wt %. When there was a further increase in the amount of the copper ions (up to 0.07 wt %), the nanocrystals formed on the scaffold surface. These phenomena were similar to our previous report, in which the synthesis and characterization of copper-substituted hydroxyapatite microspheres [20] proved that the addition of copper ions under hydrothermal conditions changed the HA ribbons into flower-like microspheres. However, with a further increase in the copper ions, the flower-like microspheres degraded and produced a considerable amount of irregular flakes. These findings proved that the addition of the copper ions did affect the morphology of the HA crystals under hydrothermal conditions. Zhang et al. observed that Mg^2+^ could adsorb onto the calcite crystal surface in a non-uniform manner and change the crystal morphology [21]. Thus, we proposed that copper ions might tend to adsorb onto the scaffold surface and further regulate the growth of the crystal nucleus, which led to the morphology change in calcium phosphate deposition.

The results implied that the micro/nano-structures deposited on the HA scaffolds could be attributed to the effect of Cu in the initial solutions. The surfaces of the scaffolds have been evaluated by controlling the Cu in the initial solutions during hydrothermal treatment, which might have triggered an EC response and increased the angiogenesis capacity.

### 3.2. In Vitro Evaluation of HA and Cu_x_-HA Scaffolds

**Figure 7:**
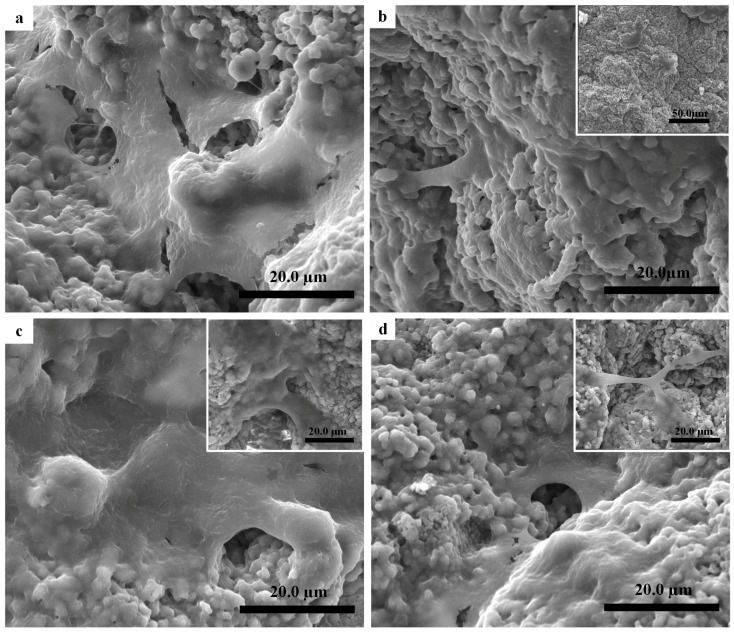
SEM images illustrated endothelial cell (EC) culture after five days: (**a**) as-sintered HA scaffold; (**b**) Cu_3_-HA scaffold sphere-like structures; (**c**) Cu_5_-HA scaffold flower-like structures; and (**d**) Cu_7_-HA nano-structed structures.

The typical EC morphologies on the surface of scaffolds incubated for five days are illustrated in Figure 7. The EC spread out completely to cover an area of 0.79 μm^2^, showing good growth on the HA surface (Figure 7a). Some cracks were seen in the EC morphology due to the adopted dehydration protocol.

The morphology of the Cu_3_-HA scaffold was sphere-like in shape, containing micro/nano-crystals; the Cu_5_-HA morphology was flower-like and also included micro/nano-crystals; and Cu_7_-HA had a nano-crystal shape. These different morphologies would affect the EC proliferations.

Fewer EC were spread on the sphere-like shape surface of the Cu_3_-HA scaffold to cover an area of 0.30 μm^2^ (Figure 7b). On the flower-like shape surface of the Cu_5_-HA scaffold (Figure 7c), more EC spread to cover an area of 0.90 μm^2^ (Figure 7c). For the nanocrystal-like surface of the Cu_7_-HA scaffold, the EC spread less than HA and Cu_5_-HA but more than Cu_3_-HA to cover an area of 0.65 μm^2^ (Figure 7d). The results imply that the changing micro/nanostructure of the scaffold could affect the EC proliferation. The flower-like structure obtained the best result.

In essence, the synergistic effect of the morphology and released ions may influence the EC proliferation. Conversely, the results implied that the distribution of EC on the Cu_5_-HA (0.05 wt %) scaffold was greater than Cu_3_-HA (0.03 wt %) and Cu_7_-HA (0.07 wt %). Thus, we inferred that the increased EC on Cu_5_-HA scaffold was dependent on their morphological features, which is related to the micro/nano-structure instead of the amount of copper released.

### 3.3. EC Viability of HA and Cu_x_-HA Scaffolds

The coatings on the HA scaffold surfaces had different micro/nano-morphologies, which changed by modulating the Cu ion concentrations in a modified hydrothermal treatment. The EC growth behavior on different groups of the scaffolds was analyzed. A sustainable increase in the EC proliferation on scaffold groups was observed with increasing culture time.

**Figure 8:**
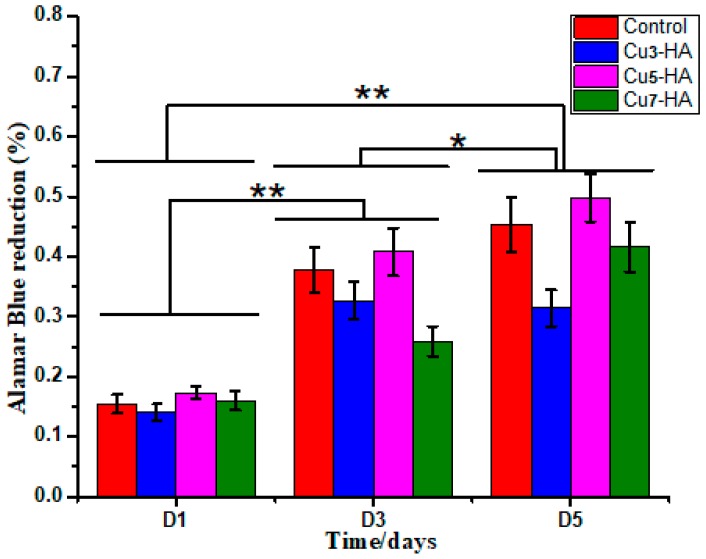
The bar chart illustrated EC viability during cell culture. * and ** denote significant differences between groups at the *p* < 0.1 and *p* < 0.05 levels, respectively.

Figure 8 illustrated that on the first and third days, the t-test value showed a significant difference between groups (*p* < 0.05). The t-test values for the first and fifth days were both statistically significant (*p* < 0.05). Overall, these results indicate that EC viability is dependent on the surface coatings of scaffolds and the culture time. Within the same group, a different observation was illustrated in the EC viability rate with different culture times (one, three and five days). After culturing for one and three days, the EC viability on Cu_5_-HA exhibited insignificant differences compared with those cultured on other groups. On the third day, the EC viability on HA and Cu_5_-HA was significantly different compared with the Cu_3_-HA and Cu_7_-HA scaffolds. Considering the statistical significance at an α-level of 0.1, Cu_5_-HA exhibited a significant difference on day five compared with Cu_3_-HA and Cu_7_-HA (*p* < 0.1), whereas the control (HA) did not significantly differ to Cu_5_-HA (*p* = 0.42).

Less EC viability was observed on Cu_3_-HA than on other groups, which confirmed the previous results for fewer EC attachments, as shown in Figure 7. Furthermore, the Cu_5_-HA scaffolds have a unique feature that is important for angiogenesis and supports bone healing when the scaffolds are used as bone substitution, especially in the earlier days after injuries. Therefore, regulating and controlling the micro/nano-structures of the HA fiber scaffolds is important.

### 3.4. In Vivo Evaluation of HA and Cu_5_-HA Scaffolds

**Figure 9:**
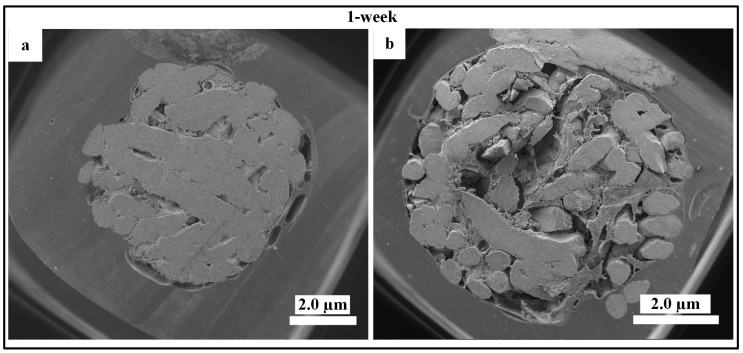
SEM high-magnification pictures characterized HA and Cu_5_-HA after one week of implantation: (**a)** no blood vessels formed on HA scaffolds and (**b**) blood vessels formed on Cu_5_-HA.

**Figure 10:**
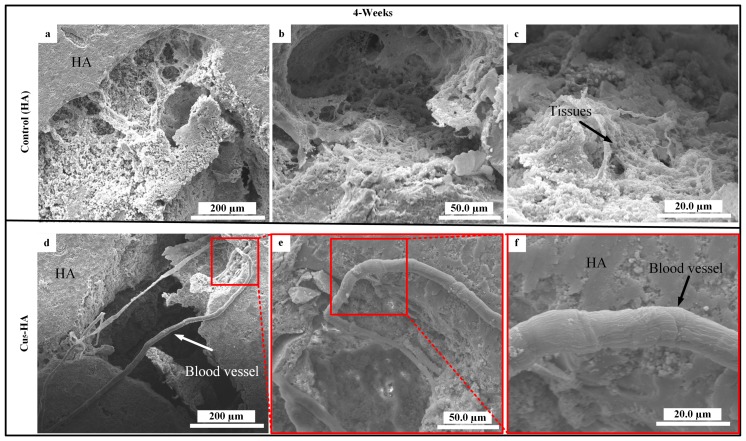
SEM high-magnification pictures characterized blood vessels on HA and Cu_5_-HA after four weeks of implantation: (**a**,**c**,**d**) no blood vessels formed on the HA scaffolds and (**d**,**e**,**f**) blood vessels formed on the Cu_5_-HA scaffold.

To examine the new biomaterial HA scaffolds (control) and Cu_5_-HA (i.e., the best result from the in vitro test), suitable skeleton skin defects in a poorly vascularized location (Figure 2c) must be established in vivo through the use of appropriate animal models. The in vivo animal model allows for the standardization or elimination of variables that contribute to the success or failure of tissue engineered materials [22]. Figure 9a,b demonstrate that no blood vessels formed on the HA and Cu_5_-HA scaffolds after one week of implantation. This can be attributed to the earlier harvesting time or limited time for blood vessel formation. Figure 10 illustrates the HA and Cu_5_-HA scaffolds after four weeks of implantation.

Figure 10a–c demonstrate that no blood vessels formed on the HA scaffolds as only some tissues were observed. However, new blood vessels formed on the Cu_5_-HA scaffolds, which grew toward the centers of the scaffold between the micropores and through the interconnecting paths, as indicated by the arrows in Figure 10d–f. We inferred that Cu_5_-HA has more bioactive material compared with the HA scaffold. The micro/nano-structure of the Cu_5_-HA scaffold resulted in more angiogenesis, which formed the new blood vessels on the other side. The absence of blood vessel formations can be attributed to the smooth surfaces of HA scaffolds.

Notably, performing the histological analysis was difficult because of the dimensionless blood vessels in the internal Cu_5_-HA scaffolds.

**Figure 11:**
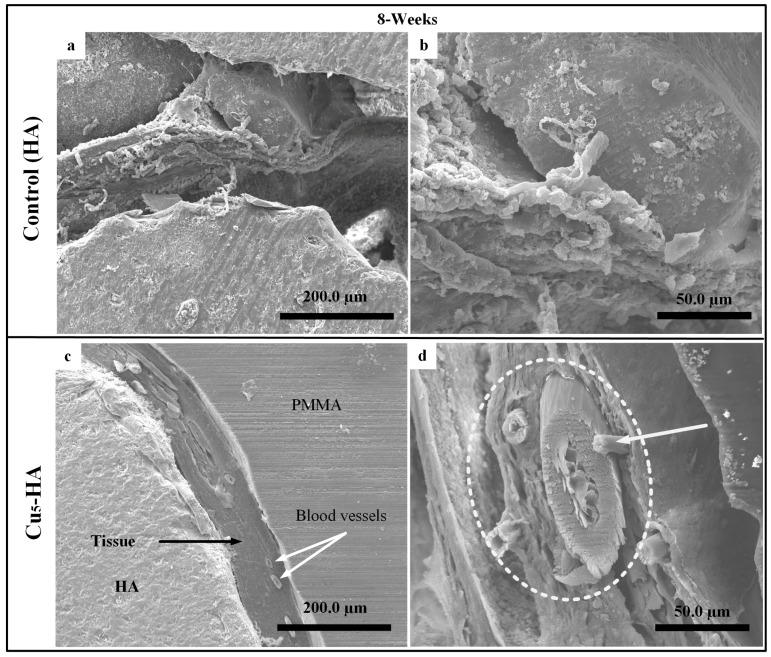
SEM images characterized the surface of HA, Cu_5_-HA after incubation for eight weeks on rabbits. (**a**,**d**) The typical morphology of HA scaffolds and (**c**,**d**) Cu_5_-HA scaffolds.

Figure 11a–d illustrate the HA and Cu_5_-HA scaffolds after eight weeks of implantation. No formation of blood vessels was observed on the HA scaffolds, as shown in Figure 11a,b. Healthy fiber muscles (aponeurosis) that were attached to the wall of the Cu_5_-HA scaffold are shown in Figure 11c,d. The muscle layers, which included the blood vessels surrounded by vascular vessels, are indicated by white arrows and circles as shown in Figure 11c,d.

**Figure 12:**
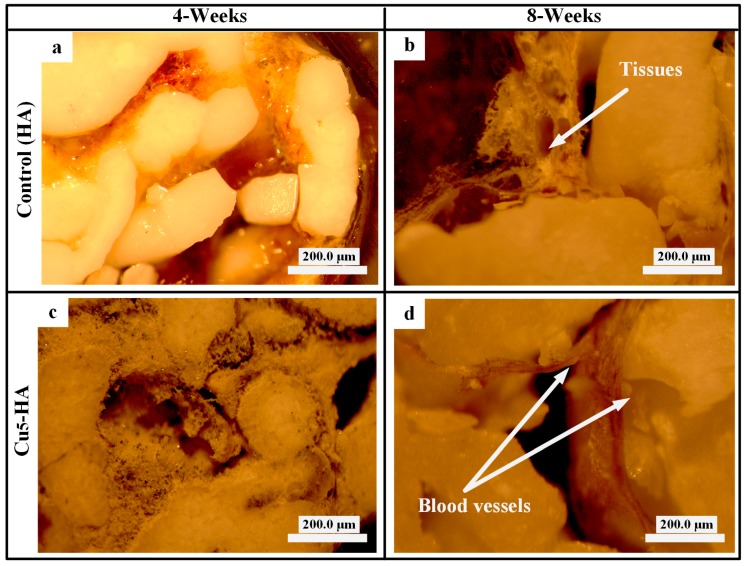
Optical microscopic characterizations of the HA and Cu_5_-HA scaffolds after eight weeks of implantation. (**a**,**b**) Tissues attached to HA scaffold and (**c**,**d**) blood vessel on Cu_5_-HA scaffolds.

Figure 12 also illustrates the optical microscopic (AxioCam Erc 5s, Carl Zeiss, Jena, Germany) comparison of the HA and Cu_5_-HA scaffolds after four and eight weeks of implantation. No blood vessels formed on the surface of the HA scaffold (Figure 12a,b) compared with the Cu_5_-HA scaffolds, which has blood vessel formation after eight weeks (Figure 12d).

In this test, the Cu_5_-HA scaffold resulted in the formation of more blood vessels compared to the HA scaffolds. The result fills the gap in the literature of biomaterials and may promote a reduction in the healing time required for bone fractures in the future, especially for critical size defects (CSD).

## 4. Conclusions

Under hydrothermal conditions, the micro/nano-structured morphologies were successfully fabricated on the surface of the HA scaffolds with the assistance of Cu. With an increase in Cu concentration, the surface micro/nano-structure of the HA scaffolds changed from being smooth to sphere-like, flower-like and finally to nano-crystals. The results suggest that Cu plays an important role in the crystal formation process on the HA scaffolds. In vitro cell cultures revealed that the surface micro/nano-structures of the HA scaffolds had an effect on EC cell proliferation. Compared with the other scaffolds, the flower-like shape was most favorable for the angiogenic proliferation of EC. In vivo tests revealed that the micro/nano-structures of the HA scaffolds influenced blood vessel formation. Compared with the control scaffold, the flower-like shape was most favorable for improving angiogenesis capacity. Although the active relationship between the surface characteristics, EC behavior and angiogenesis capacity of the flower-like structure is still unclear, the method for fabricating the micro/nano-structure on the surface of the HA scaffolds presented in this work provides a convenient surface modification approach for bone repair and regeneration.

## Figures and Tables

**Table 1:** Chemical components (% weights ± 0.05) of hydroxyapatite (HA) solutions.

Sample	Ca (%)	Cu (%)	P (%)	(Ca + Cu)/P	Ca/P	Cu/(Ca + Cu) (%)
HA	39.86	0.00	18.4	1.67	1.67	0.00
Cu_3_-HA	38.66	1.88	18.4	1.67	1.62	0.03
Cu_5_-HA	37.87	3.15	18.4	1.67	1.58	0.05
Cu_7_-HA	37.07	4.41	18.4	1.67	1.55	0.07
